# A Novel Strategy to Increase the Proliferative Potential of Adult Human β-Cells While Maintaining Their Differentiated Phenotype

**DOI:** 10.1371/journal.pone.0066131

**Published:** 2013-06-12

**Authors:** Haytham Aly, Nidhi Rohatgi, Connie A. Marshall, Tiffani C. Grossenheider, Hiroyuki Miyoshi, Thaddeus S. Stappenbeck, Scot J. Matkovich, Michael L. McDaniel

**Affiliations:** 1 Department of Pathology and Immunology Washington University in St. Louis, St. Louis, Missouri, United States of America; 2 Center for Pharmacogenomics, Department of Medicine, Washington University in St. Louis, St. Louis, Missouri, United States of America; University of Kentucky, United States of America

## Abstract

Our previous studies demonstrated that Wnt/GSK-3/β-catenin and mTOR signaling are necessary to stimulate proliferative processes in adult human β-cells. Direct inhibition of GSK-3, that engages Wnt signaling downstream of the Wnt receptor, increases β-catenin nuclear translocation and β-cell proliferation but results in lower insulin content. Our current goal was to engage canonical and non-canonical Wnt signaling at the receptor level to significantly increase human β-cell proliferation while maintaining a β-cell phenotype in intact islets. We adopted a system that utilized conditioned medium from L cells that expressed Wnt3a, R-spondin-3 and Noggin (L-WRN conditioned medium). In addition we used a ROCK inhibitor (Y-27632) and SB-431542 (that results in RhoA inhibition) in these cultures. Treatment of intact human islets with L-WRN conditioned medium plus inhibitors significantly increased DNA synthesis ∼6 fold in a rapamycin-sensitive manner. Moreover, this treatment strikingly increased human β-cell proliferation ∼20 fold above glucose alone. Only the combination of L-WRN conditioned medium with RhoA/ROCK inhibitors resulted in substantial proliferation. Transcriptome-wide gene expression profiling demonstrated that L-WRN medium provoked robust changes in several signaling families, including enhanced β-catenin-mediated and β-cell-specific gene expression. This treatment also increased expression of *Nr4a2* and *Irs2* and resulted in phosphorylation of Akt. Importantly, glucose-stimulated insulin secretion and content were not downregulated by L-WRN medium treatment. Our data demonstrate that engaging Wnt signaling at the receptor level by this method leads to necessary crosstalk between multiple signaling pathways including activation of Akt, mTOR, Wnt/β-catenin, PKA/CREB, and inhibition of RhoA/ROCK that substantially increase human β-cell proliferation while maintaining the β-cell phenotype.

## Introduction

Inadequate β-cell mass is a defect common to both types 1 & 2 diabetes (T1DM, T2DM). Although adult human β-cells have very low proliferation rates *in vivo*, significant levels of proliferation occur, for example during pregnancy and conditions of insulin resistance, indicating the existence of regulatory mechanisms. Multiple investigators have highlighted the importance of self-duplication *in vivo* as the major source of postnatal β-cell expansion although contributions from stem cells are not excluded [1]–[3]. However, *in vitro* studies by Rutti et al. found that proliferation of dispersed human β-cells is a very rare event that was not significantly enhanced using a variety of trophic factors and matrices [4]. In addition, Neilson et al. observed that intact isolated human islets remained functional for months, but did not proliferate under the culture conditions used [5]. Based on this *in vitro* proliferation barrier, there is a compelling need to identify the regulatory mechanisms and strategies that will unmask the proliferative capacity of pre-existing differentiated adult human β-cells in intact islets, and may lead to the identification of new drug targets [6].

Several studies have focused on developing strategies to expand or restore β-cell mass by exploring pathways that drive β-cell proliferation while maintaining β-cell function [7]–[14]. Using *in vitro* and *in vivo* models, delivery of transcription factors that facilitate cell cycle entry, such as hepatocyte nuclear factor-4α [14], or regulate the cell cycle including c-Myc [13], cyclin D1 [7], cyclin-dependent kinase 2 (cdk2), cyclin E [12], and cdk6 [8], [11], have been shown to continuously drive the replication of adult human β-cells.

Our previous *in vitro* studies demonstrated that engaging the Wnt/β-catenin pathway by direct GSK-3 inhibition with pharmacologic agents in combination with nutrient activation of mTOR, enhanced DNA synthesis, cell cycle progression and proliferation of adult human β-cells in a rapamycin-sensitive manner [15], [16]. Our studies also determined that human islets exhibited insulin signaling pathway resistance, as indicated by a lack of Akt phosphorylation, in comparison to rodent islets. Insulin signaling pathway resistance in human islets is due in part to mTOR/S6K1-mediated feedback inhibition of the insulin-signaling pathway that results in degradation of IRS1/2 [15], [17] and prevents growth factors from activating Akt and subsequent inhibition of GSK-3. Although treatment of human islets with inhibitors of GSK-3 circumvented insulin resistance by engaging the canonical Wnt signaling pathway, Akt signaling was not restored [18]. Surprisingly, nearly all of the isolated human islets that we receive display insulin signaling pathway resistance, although none of the islet donors were diagnosed with type 2 diabetes. The reasons for the insulin signaling pathway resistance are unclear but may be due to multiple factors including islet isolation procedures, ischemia/reperfusion injury, shipping, hypoxia, oxidative stress and inflammation [19].

Our present goal is to identify the regulatory mechanisms necessary to achieve significant proliferation of pre-existing differentiated adult human β-cells using intact cadaveric islets. This strategy is achieved by physiologically engaging Wnt signaling at the receptor level that activates both canonical and non-canonical Wnt signaling. At the cell surface, Wnt ligands bind to a G protein-coupled Frizzled (Fzd) receptor and lipoprotein receptor-like proteins, LRP5/6 co-receptor. This results in activation of diverse signaling events including β-catenin-dependent transcription (canonical pathway), and several distinct β-catenin-independent pathways (non-canonical pathways) [20].

In canonical Wnt signaling, the absence of Wnt ligands binding to surface receptors results in active GSK-3 that phosphorylates β-catenin, and targets its proteasomal degradation [21]. Upon activation of Wnt signaling, in the presence of Wnt ligands, GSK-3 is inactivated leading to cytoplasmic accumulation of active β-catenin that translocates to the nucleus [22] and associates with other transcription factors including the T-cell factor/lymphocyte enhancer factor (TCF/LEF). Overall, this cascade results in the transcription of Wnt-target genes necessary for proliferation [23].

In the non-canonical Wnt pathways, the binding of Wnt proteins to Fzd and Lrp5/6 receptors leads to the activation of the Rho and Rac GTPases, which are important regulators of cell adhesion, transcription regulation and cell cycle progression [15]. Rho GTPase is a member of the Ras superfamily of proteins that regulates cytoskeletal dynamics [24] and plays critical roles in the regulation of gene expression and cell proliferation [25]–[27]. Also associated with the Rho GTPase pathway are ROCK proteins that are involved in cytoskeletal processes including migration, apoptosis, cytokinesis, proliferation and differentiation [28].

In addition to the Wnt glycoproteins, the roof plate-specific spondin (R-spondin) proteins bind to the LRP5/6 receptor and activate Wnt signaling in association with β-catenin activation [29]. The R-spondin protein family (RSpo) consists of 4 members (RSpo 1–4) that share ∼60% sequence similarity and comparable domain organization [30]. All four RSpo proteins associate with Wnt/β-catenin signaling family members and induce proliferation of crypt epithelial cells that lead to enlargement of the mouse gastrointestinal tract [30], [31]. A recent study by Kazanskaya et al. demonstrated that R-spondin-3 is required for Wnt/β-catenin-mediated activation of vascular endothelial growth factor (VEGF) signaling [32]. Furthermore, Wong et al. demonstrated that mouse islets express R-spondin-1 protein that leads to activation of canonical Wnt signaling and increased β-cell proliferation and insulin secretion [33].

In addition to Wnt and mTOR signaling, the cyclic AMP-dependent protein kinase A (PKA) pathway also plays an important role in the regulation of gene expression, function, and proliferation of β-cells [34]. PKA mediates inhibition of GSK-3 by two independent mechanisms: a direct phosphorylation of GSK-3 [34] and by activation of Akt that inhibits GSK-3 [35]–[38]. In addition, stimulation of PKA by cAMP leads to the phosphorylation of cyclic AMP response element binding protein (CREB) at Ser133 that enhances the transcription of proliferation-related genes [39]. GSK-3β regulates CREB activity by phosphorylating CREB at Ser129 [40] and increases its transcriptional activity [33], [41].

Here we utilized a system to deliver Wnt and R-spondin ligands to cultured cells. This system involves the use of conditioned medium from L-cells that constitutively produce Wnt3a, R-spondin-3 and Noggin. This system has been successfully used to grow mouse colonic epithelial cells that are enriched for stem cells. Our goal was to test if this system could be adapted to human islet cells in culture. We found that this unique conditioned medium has allowed us to significantly increase proliferation while maintaining differentiation of adult human β-cells in intact islets. This novel strategy engages both canonical and non-canonical Wnt signaling, and in combination with RhoA/ROCK inhibition removes insulin signaling pathway resistance and allows necessary crosstalk between Wnt/GSK-3/β-catenin, Akt/mTOR, and PKA/CREB pathways associated with human β-cell regenerative processes.

## Materials and Methods

### Materials

Lithium chloride (LiCl) and ROCK inhibitor (Y-27632) were from Sigma. Connaught Medical Research Laboratories (CMRL) 1066 media, penicillin, streptomycin, Hank's balanced salt solution (HBSS) were from Invitrogen. PKA inhibitor, [N-[2-(*p*-Bromocinnamylamino)ethyl]-5-isoquinolinesulfonamide. 2HCl] (H-89) and rapamycin were from Enzo Life Sciences International, Inc. TGF-β type 1 receptor inhibitor (SB-431542) was from Calbiochem. Akt inhibitor XII was from Millipore. Defined fetal bovine serum (FBS) was from Hyclone. ^3^H-thymidine-aqueous, 2 Ci/mmol, 1 mCi/ml was from Perkin-Elmer and American Radiolabeled Chemical, Inc. All primary antibodies used were from Cell Signaling. The peroxidase-conjugated secondary antibodies were obtained from Jackson ImmunoResearch laboratories. All other chemicals were from commercially available sources.

### Ethics Statement

Isolated cadaver-derived human islets were obtained from the Integrated Islet Distribution Program (IIDP) sponsored by the NIDDK and the JDRFI and the JDRFI Sponsored Islets for Research Program at Washington University (JDRF-31-2008-382). The IIDP uses only cadaver donors that have consented to research. The Washington University Medical Center (WUMC) Human Studies Committee (HSC) IRB approved all studies involving the use of isolated cadaver-derived human islets (Approval number: 93-0068). The IRB exempted the study from HIPAA compliance based on regulatory definition of human subject. Review date: 7/8/2004; review committee: 08 MRCR.

### Human Islets

Upon arrival, the islets were collected by centrifugation, cleaned and hand-selected in cCMRL under a stereomicroscope and treated immediately or after overnight culture at 37°C. Human islet donor data: 26 donors; 9 males, 12 females and 5 not documented; age, mean 41.8 yrs (range 17–65); BMI, mean 28.7 (range 22.3–38.4); purity, mean 85.8% (range 50–95%); and viability, mean 92.9% (range 85–95%). None of the donors were diagnosed with diabetes.

### Conditioned Media

Wnt3a, R-spondin-3 and Noggin (L-WRN) conditioned medium based on CMRL (Invitrogen) supplemented with 10% FBS was constructed as previously described [42]. Briefly, L-WRN conditioned medium was prepared from mouse L cells stably expressing Wnt3a, cells transfected with R-spondin-3 and Noggin expression vector and doubly-selected in media containing hygromycin and G418 (Sigma). L-WRN+ conditioned medium was prepared by adding 10 µM Y-27632 and 10 µM SB-431542 inhibitors to L-WRN conditioned medium. Conditioned medium collected from wild type L-cells was used as a control.

### ^3^H-Thymidine Incorporation

Islets (100) were cultured in Petri dishes (35×10 mm) for 4 days in 1 ml CMRL (5 or 8 mM glucose, 10% FBS, 2 mM L-glutamine, 100 units/ml penicillin, 100 µg/ml streptomycin) containing treatment conditions, as indicated in the figure legends, at 37°C, under 95% air/5% CO_2_. ^3^H-thymidine was added to each dish at a final concentration of 10 µCi/ml during the last 24 h. The incorporation of ^3^H-thymidine into DNA was determined by trichloroacetic acid precipitation and scintillation counting.

### Quantitative Real Time RT-PCR

Islets (100) were cultured for various times in 1 ml of CMRL (5, 8 or 20 mM glucose, 10% FBS, 2 mM L-glutamine, 100 units/ml penicillin, 100 µg/ml streptomycin) under different treatment conditions, as indicated in the figure legends, at 37°C, under 95% air/5% CO_2_. The total RNA was extracted using Qiagen RNAeasy kit (Qiagen) according to the manufacturer's instructions. First-strand cDNA was synthesized from 0.5–1 µg of total RNA using a High Capacity cDNA Reverse Transcription kit (Applied Biosystems). The expressions of the analyzed genes were investigated using pre-designed TaqMan primers/probes (Applied Biosystems), and all probes were 6-carboxyfluorescein (FAM™) fluorescently labeled with a 3′ non-fluorescent quencher, MGB. 100 nanograms of reversed-transcribed total RNA and Taqman Gene Expression MasterMix in a total volume of 20 µL PCR reaction was performed using StepOnePlus Real-Time PCR system (Applied Biosystems). Amplifications for each gene were carried out in triplicate, and mean values were normalized to the mean value of the endogenous control, β-actin. The relative fold changes of the analyzed genes were calculated using the comparative threshold cycle method (2^−ΔΔCt^) [43].

### RNA-Sequencing

Two islet preparations (from different donors) were subjected to RNA-sequencing; within each preparation, subsets of islets were treated with 5 or 8 mM glucose, 8 mM glucose+5 mM LiCl or 8 mM glucose together with L-WRN+ for 48 h immediately after initial receipt and hand-selection. Polyadenylated mRNA was obtained from 1 µg of human islet total RNA using oligo(dT)-coupled Dynabeads (Invitrogen), and mRNA-sequencing library preparation was performed essentially as described [44], [45], using Illumina HiSeq 2000 sequencers and 3′ library indexing rather than 5′ bar-coding. Raw sequence files have been deposited in the NCBI sequence read archive (SRA) with the accession ID SRA072806 (http://www.ncbi.nlm.nih.gov/sra/?term=SRA072806). The ‘transcriptome-only’ alignment function of TopHat 1.4.0 [46] was used together with the Ensembl GRCh37 human genome sequence and associated transcriptome map (gtf) provided through the Illumina iGenomes project (download available at http://tophat.cbcb.umd.edu/igenomes.html) to align 50 nt single-end sequencing reads. The aligned read count for each sequencing library (i.e. treated islet sample) was 8.5±0.7 million (mean ± standard error of the mean).

### Filtering of RNA-Sequencing Data, Differential Gene Expression, and Categorization

The DESeq package [47] was used to normalize read depth across different sequencing libraries and to calculate fold-changes between treated islet samples. Expression of each mRNA is reported as the normalized number of reads mapped to each mRNA. mRNAs with fewer than 8 reads mapping in any sample (i.e. less than 1 millionth of the total number of reads) were removed from further consideration. Those mRNAs regulated in response to L-WRN+ treatment were defined as those with at least a 1.5 fold-change (in either direction) between L-WRN+ and 8 mM glucose-treated islets from the same donor, and for which the same degree and direction of fold-change was observed in islet preparations from both donors. Partek Genomics Suite 6.6 (Partek, St. Louis, MO) was used to perform unsupervised hierarchical clustering with Euclidean dissimilarity and average linkage, and to derive heatmaps. The MetaCore online suite of gene network analytic tools (Thomson Reuters) was used to interrogate regulated mRNAs for membership in Gene Ontology categories and custom MetaCore network groups. Pathway diagrams in MetaCore were prepared with the restriction that genes displayed had to be present in human islets.

### Insulin Secretion and Content

Islets were cultured for 5 days in CMRL-1066 medium containing 10% FBS, 8 mM glucose or in L-WRN±10 µM RhoA/ROCK inhibitors as indicated in the figure legend. At the end of 5 days, islets were incubated for 2 h or 24 h in CMRL containing 5 mM glucose and then four aliquots of 10 islets were counted for each treatment group. Islets were preincubated for 30 min in CMRL (5 mM glucose), and media was replaced with CMRL with 5, 16.5 mM glucose, or 16.5 mM glucose+10 µM forskolin for 1 h. Insulin secreted in the culture supernatant was assayed by Immulite solid-phase competitive chemiluminescent enzyme immunoassay kit from Siemens Healthcare Diagnostics Inc. Islet insulin content was measured in separate batches of 20 islets after ethanol/acid (77% ethanol, 1% hydrochloric acid) extraction. Insulin content from three aliquots of 20 islets was also measured at the start of the incubation period (Time 0). Islet proteins were extracted using RLT extraction buffer (Invitrogen) and quantified with BioRad Bradford reagent. The insulin values were normalized for the total protein content extracted from the same islets.

### Western Blot Analysis

Groups of 150–200 islets were incubated in CMRL medium and treated as indicated in the figure legends. Islet proteins were prepared using the Nuclear Extract Kit (Active Motif) according to the instructions of the manufacturer. Protein concentrations were measured using the BioRad Bradford reagent. Fifteen micrograms of each sample were loaded in each lane of a 4–12% Bis-Tris polyacrylamide gel (Invitrogen). Proteins were transferred to a nitrocellulose membrane, and the membrane blocked with 5% nonfat milk for 1 h, incubated with the antibody (1∶1000) against the desired protein overnight at 4°C, washed, incubated with the appropriate secondary antibody (1∶5000) for 1 h at room temperature, washed again, and developed using ECL (Amersham) or Immun-Star WesternC (BioRad).

### Immunohistochemistry

Human islets (65) were treated for 5 days, washed with cold PBS and then dissociated into single-cell suspension by incubation in 1 mL Accutase (Innovative Cell Technologies) at room temperature for 15–30 min and dispersed by gentle pipetting. Single-cell suspensions were cytospun onto coated slides (3000–4000 cells/slide). Cells were fixed in 4% paraformaldehyde (Electron Microscopy Sciences) for 30 min, permeabilized using 1% triton X-100 for 20 min, and blocked with 3% BSA for 1 h at room temperature. Indirect immunofluorescence was performed using primary antibodies, rabbit anti-insulin (1∶100; Cell Signaling), mouse anti-Ki-67 (1∶100; Dako), and secondary antibodies, Alexa 488 goat anti-rabbit (1∶1000) and Alexa 555 goat anti-mouse (1∶1000) (Molecular Probes). Nuclei were counterstained with 4′, 6′- diamindino-2-phenylindole (DAPI; Molecular Probes). All microscopy was performed using a Nikon TE300 microscope equipped with CoolSNAP ES2 camera (Photometrics); images were acquired using the MetaMorph software package (Molecular Devices) with 1×1 binning and 128 pixels×128 pixels imaging field.

### Expression of Data and Statistics

All data are expressed as means ± standard error (SE). Statistical analyses were performed using unpaired t tests or one-way ANOVA followed by Newman-Keuls multiple comparison test. Significant differences are indicated by *P<0.05, **P<0.01, ***P<0.001.

## Results

### Activation of the Wnt signaling pathway with Wnt3a/R-spondin-3, Noggin containing conditioned media (L-WRN) and RhoA/Rock inhibitors (L-WRN+)

**Figure 1:**
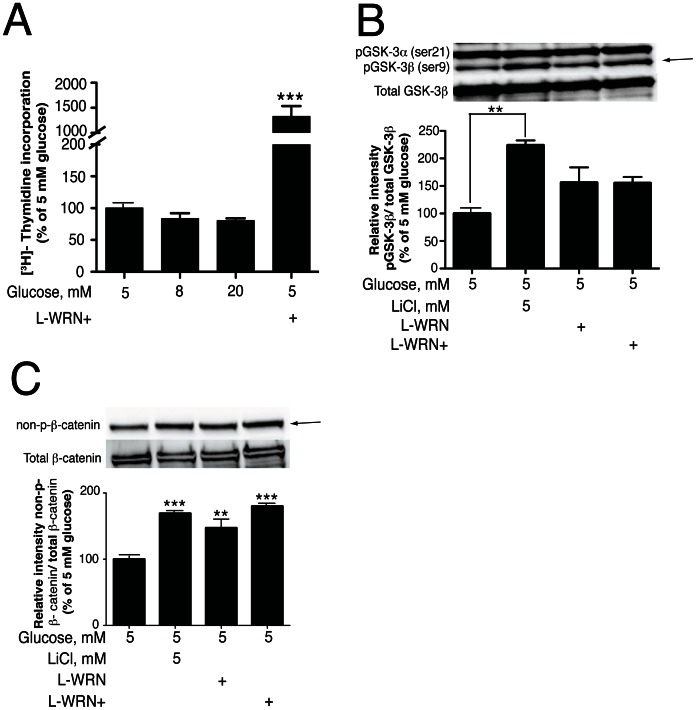
L-WRN conditioned medium activates the Wnt/β-catenin signaling pathway. (A) 100 islets were cultured for 4 days in CMRL-1066 medium containing 10% FBS and 5, 8 or 20 mM glucose or in 50% Wnt3a, R-spondin-3 and Noggin conditioned medium (L-WRN)+Y-27632 and SB-431542 inhibitors. ^3^H-thymidine was added 24 h before the end of the 4-day period. (B) & (C): islets (150) were treated for 24 h and 72 h, respectively, in CMRL-1066 medium containing 10% FBS and 5 mM glucose ± 5 mM LiCl or in 50% L-WRN ± Y-27632 and SB-431542 inhibitors. Samples were processed for Western blotting and quantitated by Image Lab™ software. pGSK-3β and non-p-β-catenin proteins were normalized to total GSK-3β and total β-catenin proteins, respectively. Data are the means ± SE of n = 3. *P<0.05, **P<0.01 and ***P<0.001 denote significant differences between the treatment condition and the control.

As a novel method to stimulate Wnt signaling, in human islet cells, we utilized 50% conditioned medium from L-cells that constitutively express Wnt3a, R-spondin-3 and Noggin (L-WRN) [42]. This experimental system was previously developed to expand the stem cell population of cultured mouse colon epithelial cells [42]. To augment cell survival we additionally added ROCK and RhoA inhibitors [48], [49]. We found that the L-WRN+ medium (containing RhoA and ROCK inhibitors) stimulates a striking level of DNA synthesis in islet cells cultured in 5 mM glucose. In contrast elevated levels of glucose did not stimulate DNA synthesis (Figure 1A). Thus the L-WRN medium plus inhibitors can overcome the well-known resistance of cultured islet cells to mitotic insulin signaling [16]–[18], [50], [51].

Previously, we determined that inhibition of GSK-3 with LiCl, to engage Wnt signaling, resulted in a significant increase in DNA synthesis [6]. In Figure 1B, we compared activation of downstream targets by LiCl and L-WRN conditioned medium. Treatment of human islets for 24 h with LiCl resulted in a significant increase in pGSK-3β (Ser9, arrow). However, L-WRN and L-WRN+ treatment did not significantly increase pGSK-3β (Ser9). Active GSK-3β (non-phosphorylated) destabilizes and degrades β-catenin by phosphorylation of Ser33, Ser37, and Thr41 [52]. In Figure 1C, treatment of human islets for 24 h with L-WRN conditioned medium (plus or minus RhoA/ROCK inhibitors) or LiCl significantly enhanced the accumulation of non-phosphorylated β-catenin protein (active form) in human islets (arrow).

These results suggest that, in addition to the effects on Wnt activation, the conditioned media may also enhance the accumulation of active β-catenin *via* a Wnt-independent mechanism [30], [53], [54].

### mTOR dependency of L-WRN+ conditioned media-stimulated DNA synthesis in human islets

**Figure 2:**
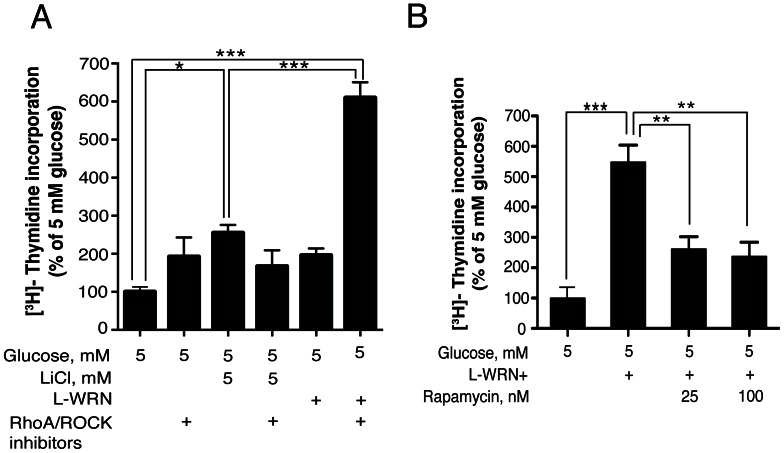
Activation of Wnt and inhibition of RhoA/ROCK signaling significantly increase DNA synthesis in human islets and is mTOR-dependent. (A & B) Islets (100) were cultured for 4 days in CMRL-1066 medium containing 10% FBS and 5 mM glucose or in 50% Wnt3a, R-spondin-3 and Noggin conditioned medium (L-WRN) ± 5 mM LiCl ± Y-27632 and SB-431542 inhibitors ±25 or 100 nM rapamycin as indicated. ^3^H-thymidine was added 24 h before the end of the 4-day period. Data are the means ± SE of n = 3. *P<0.05, **P<0.01 and ***P<0.001 denote significant differences between the treatment condition and the control.

In Figure 2A, treatment of human islets for 4 days with LiCl resulted in a significant increase (∼2.5 fold) in DNA synthesis compared to 5 mM glucose (lanes 1 vs. 3). Treatment with L-WRN conditioned medium or RhoA/ROCK inhibitors alone, or in combination with LiCl did not significantly increase DNA synthesis above 5 mM glucose. However, treatment with L-WRN+ significantly enhanced DNA synthesis ∼6 fold above that of 5 mM glucose (lanes 1 vs. 6). Figure 2B shows that treatment of human islets with rapamycin 25 or 100 nM significantly inhibited DNA synthesis induced by L-WRN+, indicating that L-WRN+ mediated DNA synthesis is mTOR-dependent.

### L-WRN+ treatment increases β-cell proliferation and increases expression of β-cell specific genes

**Figure 3:**
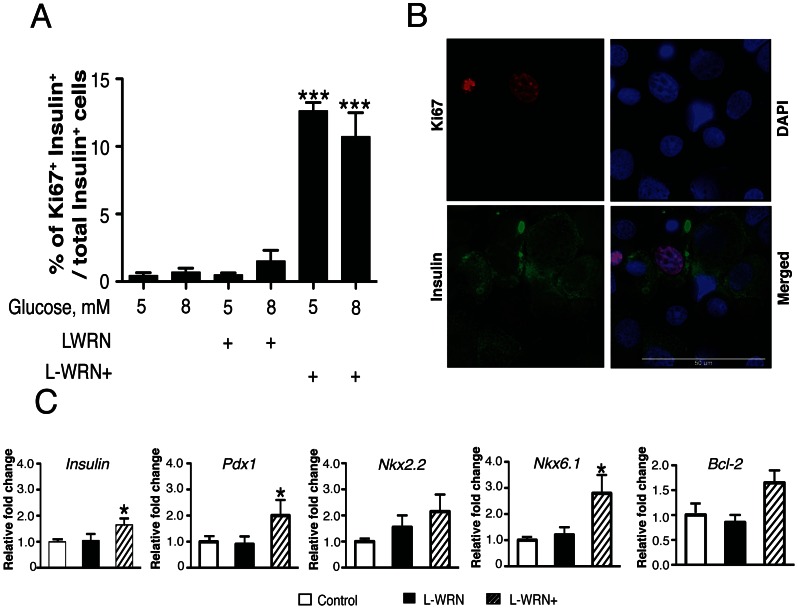
Activation of Wnt and inhibition of RhoA/ROCK signaling significantly increase proliferation of adult human β-cells. Islets (65) were cultured for 5 days in CMRL-1066 medium containing 10% FBS and 5 or 8 mM glucose or in 50% Wnt3a, R-spondin-3 and Noggin conditioned medium (L-WRN) ± 5 or 8 mM glucose ± Y-27632 and SB-431542 inhibitors as indicated. Samples were processed for immunohistochemistry as described in Materials and Methods. (A) Quantitative measurements of the % of Ki-67^+^and insulin^+^ cells/total insulin^+^ cells. For each experimental treatment 4500–7500 β-cells were counted in three independent experiments. (B) Representative immunofluorescence image of human islets treated for 5 days with L-WRN plus Y-27632 and SB-431542 inhibitors then dispersed into single cells and stained. Pink nucleus, Ki-67^+^; green, insulin^+^; and blue, nuclei stained with DAPI. (C) 75 islets were cultured for 8 h in CMRL-1066 medium containing 10% FBS and 5 mM glucose or in 50% Wnt3a, R-spondin-3 and Noggin conditioned medium (L-WRN) ± Y-27632 and SB-431542 inhibitors as indicated. Samples were processed for qRT-PCR and levels of mRNA for *Insulin*, *Pdx1*, *Nkx2.2*, *Nkx6.1* and *Bcl2* were determined as described in Materials and Methods. Transcripts were normalized to β-actin at the corresponding time point. Data are the means ± SE of n = 3. *P<0.05, **P<0.01 and ***P<0.001 denote significant differences between the treatment condition and the control.

The ability of L-WRN+ to enhance the proliferation of human β-cells in intact islets was next determined. Human islets were treated with 5 or 8 mM glucose, L-WRN or L-WRN+ for 5 days followed by dispersion of islets into single cells and stained for Ki-67 and insulin to quantify β-cell proliferation. Figure 3A demonstrates that L-WRN in the presence of 5 or 8 mM glucose did not significantly increase the number of Ki-67^+^/insulin^+^ cells compared with 5 or 8 mM glucose alone. However, culturing with L-WRN+ significantly increased Ki-67^+^/insulin^+^ cells ∼20-fold above glucose alone (lanes 1 vs. 5 or 2 vs. 6). Figure 3B is a representative immunofluorescence image of dispersed human islet cells, previously treated for 5 days with L-WRN+ at 5 mM glucose, showing colocalization of Ki-67^+^ (red nuclei) and insulin^+^ (green cytoplasm) β-cell. All nuclei are identified by DAPI staining (blue). Since similar effects were observed at both 5 and 8 mM glucose, the dramatic increase in β-cell proliferation in response to L-WRN+ was not glucose-dependent.

Due to the significant effects of L-WRN+ on increasing DNA synthesis in human islets and proliferation in human β-cells, we determined if L-WRN+ treatment affected mRNA expression of key β-cell specific transcription factors assayed by qRT-PCR. Incubation of islets for 8 h with L-WRN+ increased gene expression of *Insulin*, *Pdx1*, *Nkx2.2* and *Nkx6.1* (Figure 3C). The anti-apoptotic gene *Bcl-2* was also increased, however not significantly. These results suggested that in addition to significantly increasing DNA synthesis and β-cell proliferation, L-WRN+ treatment may exert positive effects on the maintenance of β-cell phenotype and survival of human islets through the up-regulation of key transcription factors.

**Figure 4:**
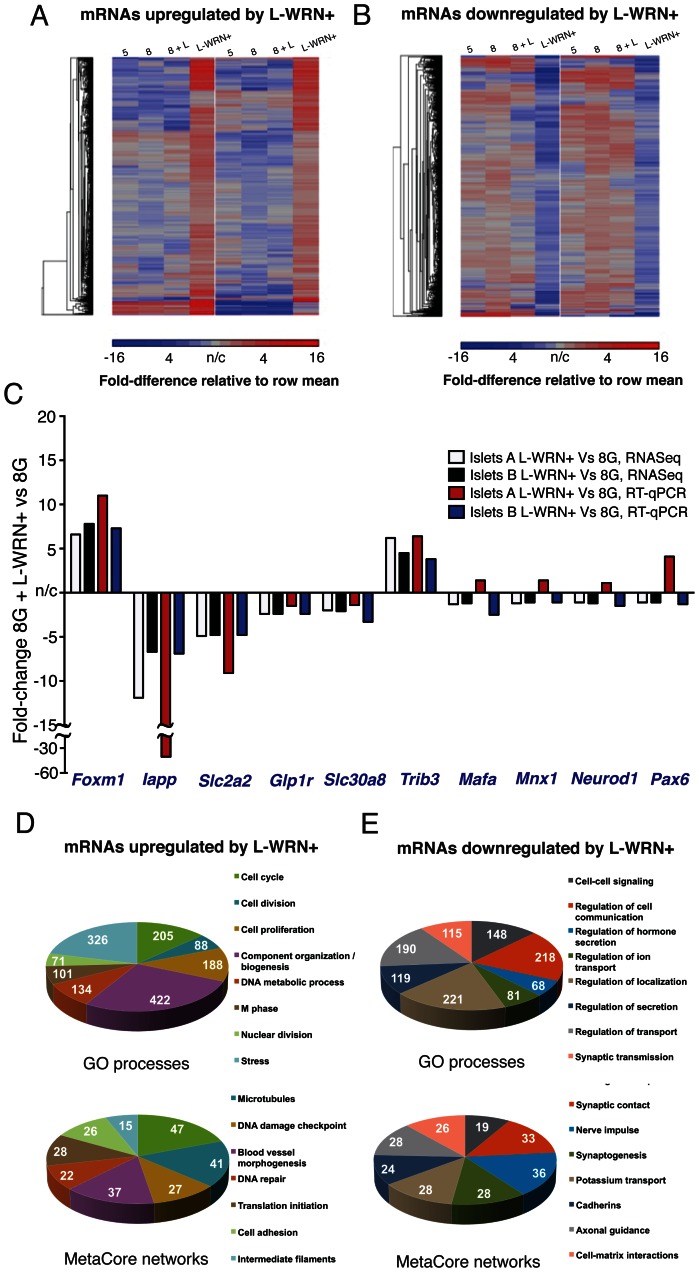
Treatment of human islets with L-WRN+ regulates large numbers of proliferative genes. (A) mRNAs upregulated in response to L-WRN+ by 1.5-fold vs treatment with 8 mM glucose alone, in islets sourced from two different donors. Relative mRNA levels in 5 mM glucose (5), 8 mM glucose (8), 8 mM glucose+LiCl (8+L) and L-WRN+-treated islet aliquots are shown; blue indicates lower abundance, red indicates higher abundance. (B) as for (A), but showing those mRNAs downregulated by 1.5-fold in response to L-WRN+ treatment. (C) qRT-PCR validation of six regulated and four nonregulated mRNAs (*Mafa*, *Mnx1*, *Neurod1*, *Pax6*); fold-changes of L-WRN+ treatment vs 8 mM glucose are shown. Individual gene expression values were normalized to the geometric mean of *B2m*, *Gapdh*, *Hmbs* and *Ywhaz* [70]. White and black bars, fold-changes observed with RNA-sequencing; red and blue bars, fold-changes observed with qRT-PCR. (D, upper) Classification of upregulated genes into Gene Ontology categories; the most significantly over-represented categories are shown. (D, lower) Classification of upregulated genes into MetaCore custom-defined categories. (E, upper and lower) as for (D), but showing downregulated genes. Further details of Gene Ontology and MetaCore assignments are provided in Tables S2 and S3.

To further explore pro-β-cell and pro-survival effects of L-WRN+, we subjected two independent preparations of human islets to whole-transcriptome, quantitative gene expression assays *via* RNA-sequencing. In response to 48 h L-WRN+ treatment, transcripts for 974 genes were enriched by at least 1.5-fold, while transcripts for 991 genes were diminished by at least 1.5-fold (from a total of 15,599 reliably detected) in comparison to islet aliquots from the same donors cultured at the same glucose concentration in standard medium (Figure 4A and 4B, Table S1). Elevation of glucose from 5 to 8 mM, or co-treatment with LiCl at 8 mM glucose, led to minimal alterations in the mRNAs strongly regulated by L-WRN+ (Figure 4A and 4B); only 175 transcripts of the total 1,965 regulated by L-WRN+ were altered by the change of glucose from 5 to 8 mM (Table S1). Genes encoding enriched transcripts were significantly over-represented in Gene Ontology processes of cell cycle regulation and cytoskeletal rearrangements related to mitosis; upregulation of such processes was expected from our data on L-WRN+-stimulated proliferation measured by Ki-67 staining (Figure 4D, Table S2). Genes encoding transcripts that were diminished in L-WRN+ treated islets were represented by categories including ion transport, cell adhesion and development (Figure 4E, Table S3). We investigated a large number of islet or β-cell-specific genes and observed regulation in a direction suggestive of increased differentiation for several of these in response to L-WRN+ (Table S4); however, a number of genes were altered in a manner resembling changes observed in disease, e.g. downregulation of *Slc2a2*/Glut2, *Glp1r*, and *Slc30a8* (ZnT8), as also shown in Figure 4C. These data demonstrate that L-WRN+ stimulates transcriptional programs in human islets that activate proliferation without causing substantial de-differentiation of β-cells, although the transcriptional profile of these β-cells cannot be said to be entirely normal.

**Figure 5:**
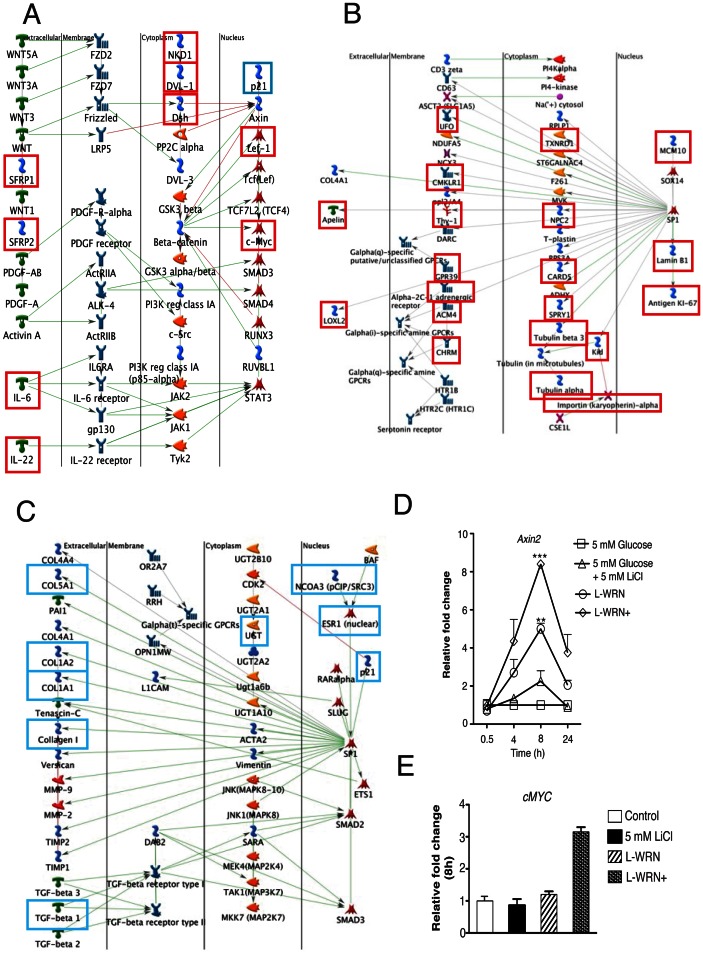
Inclusion of L-WRN+-regulated mRNAs in Wnt-related, proliferative and TGF-β signaling networks. (A) Partial network of Wnt signaling; red squares designate mRNAs upregulated by at least 1.5-fold in response to L-WRN+. (B) Proliferative signaling network focused on Ki-67 and Sp1. (C) Partial network of TGF-β signaling. Up and downregulated mRNAs in panels A–C were defined using RNA-sequencing; red squares designate mRNAs upregulated by at least 1.5-fold in respone to L-WRN+; blue squares designate downregulated mRNAs. Green connecting lines indicate activation, red connecting lines indicate inhibition, and gray lines indicate mixed or uncertain effects, according to previously published studies contained in the curated MetaCore database. (D & E) Wnt pathway targets *Axin2* and *c-Myc* compared amongst treated islets using qRT-PCR. Islets (75) were cultured for indicated times in CMRL-1066 medium containing 10% FBS and 5 mM glucose ± 5 mM LiCl or in 50% Wnt3a, R-spondin-3 and Noggin conditioned medium (L-WRN) ± Y-27632 and SB-431542 inhibitors as indicated. Samples were processed for qRT-PCR and levels of mRNA for *Axin2 and c-Myc* were determined as described in Materials and Methods. Transcripts were normalized to β-actin at the corresponding time point. Data are the means ± SE of n = 3 biological replicates. *P<0.05, **P<0.01 and ***P<0.001 denote significant differences between the treatment condition and the control.

We hypothesized that the beneficial effects of L-WRN+ could be due, at least in part, to increased expression of components of the Wnt signaling pathway in response to chronic exposure to secreted Wnt3a and R-spondin-3. Indeed, two of the signaling networks populated by enriched transcripts included the Wnt pathway (Figure 5A) (i.e. *Sfrp1*, *Sfrp2*, the Dishevelled homolog *Dvl1*, the Dvl-binding protein *Nkd1* and the Tcf-family transcription factor, *Tcf7l2*) together with a Ki-67- and Sp1-centric network) (Figure 5B). As expected, several members of TGF-β related signaling networks were diminished in representation in response to L-WRN+, including *Tgf-β* itself, *p21*/*Cdkn1a* and collagens (Figure 5C). The enrichment of *Axin2* and *c-Myc*, two downstream targets of Wnt, was validated in separate preparations of human islets using qRT-PCR, and was demonstrably higher in response to L-WRN+ than with LiCl alone or L-WRN (without RhoA/ROCK inhibitors) (Figures 5D & 5E).

### L-WRN and L-WRN+ promote crosstalk between Wnt and PKA signaling

**Figure 6:**
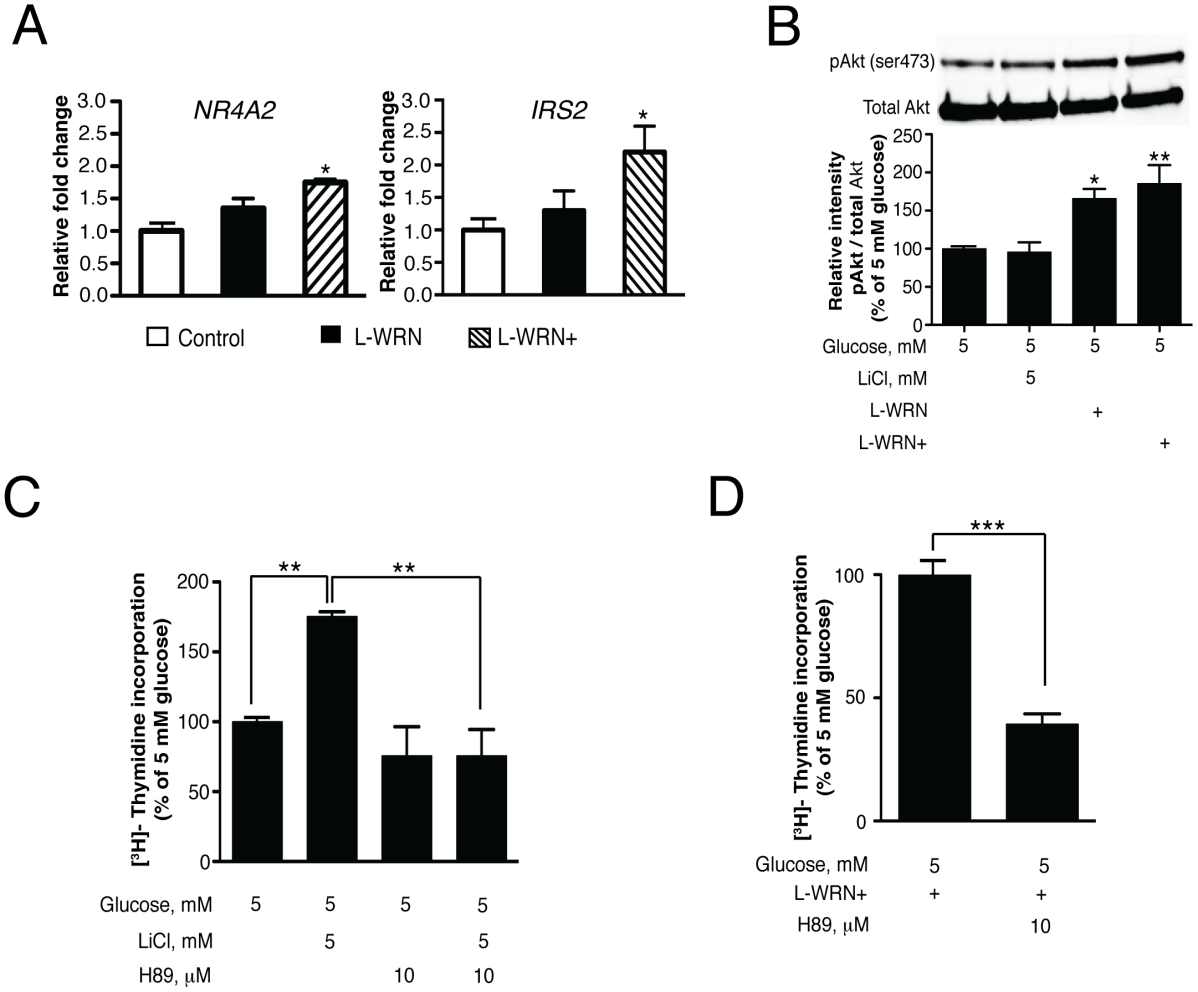
L-WRN+ promotes crosstalk between Wnt and PKA/CREB and Akt signaling in human islets. (A) Islets (75) were incubated for 8 h in CMRL-1066 medium containing 10% FBS and 5 mM glucose or in 50% Wnt3a, R-spondin-3 and Noggin conditioned medium (L-WRN)+Y-27632 and SB-431542 inhibitors as indicated. The levels of mRNA for *Nr4a2* and *Irs2* were determined as described in Material and Method. The values represent the –fold change due the indicated treatment over 5 mM glucose control treatment. (B) Islets (150) were treated for 24 h in CMRL-1066 medium containing 10% FBS and 5 mM glucose ± 5 mM LiCl or in 50% Wnt3a, R-spondin-3 and Noggin conditioned medium (L-WRN) ± Y-27632 and SB-431542 inhibitors. Samples were processed for Western blotting and quantitated by Image Lab™ software. pAkt protein was normalized to total Akt protein. Data are the means ± SE of n = 3. *P<0.05 and **P<0.01 denote significant differences between the treatment condition and respective control. (C) & (D) islets (100) were cultured for 4 days as indicated. ^3^H-thymidine was added 24 h before the end of the 4-day period. Data are the means ± SE of n = 3 with duplicate samples in each experiment. **P<0.01 and ***P<0.001 denote significant differences between the bracketed condition.

Our previous studies showed that chronic activation of mTOR/S6K1 leads to inhibition of the insulin signaling pathway and reduced Akt phosphorylation. These findings also showed that direct inhibition of GSK-3 by LiCl enhanced proliferation, but did not restore the insulin-signaling pathway [18]. CREB is an essential transcription factor for *Irs* gene expression [55]. In the current study, we examined the effects of L-WRN or L-WRN+ on expression of the CREB target genes *Nr4a2* and *Irs2*, and Akt phosphorylation. Figure 6A indicates that treatment of human islets for 8 h with L-WRN+ increased CREB activity as indicated by an increase in *Nr4a2* and *Irs2* gene expression. Importantly, Figure 6B shows that activation of Wnt signaling at the level of GSK-3 inhibition by LiCl had no effect on enhancing Akt phosphorylation (lanes 1 vs 2), whereas engaging Wnt signaling at the receptor level with L-WRN or L-WRN+ resulted in significant increases in Akt phosphorylation at Ser473 (Figure 6B lanes 1 vs. 3 and 4). These results suggest that engaging Wnt signaling with L-WRN or L-WRN+ treatment reduces insulin signaling pathway resistance, whereas direct inhibition of GSK-3 with LiCl does not. Since PKA signaling is known to be important for β-cell gene expression and proliferation, we determined whether PKA was involved in L-WRN+ or LiCl-mediated DNA synthesis. H-89, an inhibitor of PKA, significantly blocked increases in DNA synthesis in response to LiCl (Figure 6C) and L-WRN+ (Figure 6D). Overall, these results suggest that L-WRN+ promotes crosstalk between PKA, Wnt/β-catenin and Akt signaling in human islets to enhance proliferative processes.

### Activation of cAMP/PKA/CREB signaling pathway engages both canonical Wnt/β-catenin and IRS2/Akt pathways in human islets

**Figure 7:**
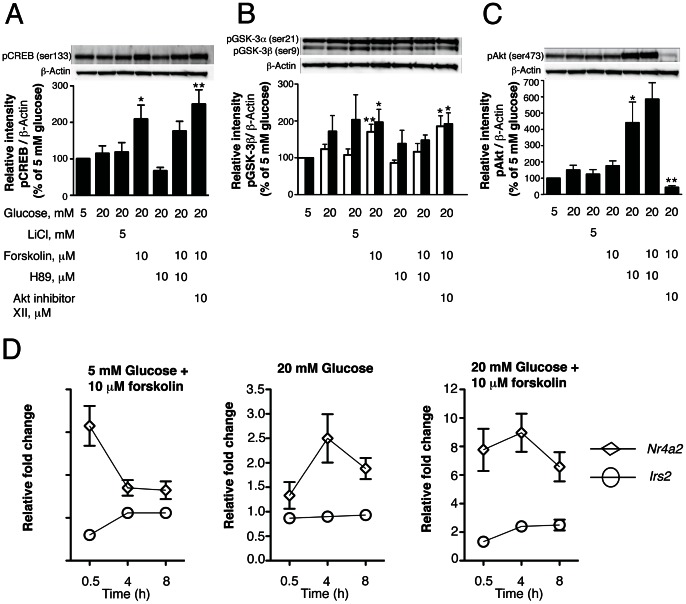
Activation of cAMP/PKA/CREB engages canonical Wnt and IRS2/Akt signaling in human islets. (A) & (B) & (C) Human islets (150) were treated for 3 h with CMRL-1066 medium containing 10% FBS, 5 or 20 mM glucose, ± 10 µM forskolin ±5 mM LiCl as indicated. The PKA inhibitor (H-89, 10 µM) and Akt inhibitor (Akt inhibitor XII, 10 µM) were added to the culture where indicated 1 h before stimulation. Cell lysates were prepared and analyzed for CREB-, Akt- and GSK-3 phosphorylation using specific antibodies as described in Materials and Methods. In (B) open columns represent pGSK-3α and black columns represent pGSK-3β. (D) Human islets (75) were incubated for 30 min, 4 and 8 h with 5 or 20 mM glucose, ±10 µM forskolin. The levels of mRNA for *Nr4a2* and *Irs2* were determined as described in Materials and Methods. The values represent the fold change due to the indicated treatment over 5 mM glucose control treatment. Data are the means ± SE of n = 3 experiments. *P<0.05 **P<0.01 and ***P<0.001 denote significant differences between treatment conditions and the respective control (5 mM glucose).

Previous studies in NIH 3T3 kidney and HEK293 fibroblast rat cell lines have shown that increasing intracellular levels of cAMP induces PKA-dependent phosphorylation and inactivation of GSK-3 [35]. Here, we show that treatment of human islets with the cell-permeable activator of adenylyl cyclase, forskolin, significantly increased CREB phosphorylation (Figure 7A) and significantly induced phosphorylation of GSK-3α and GSK-3β at Ser21 and Ser9, respectively (Figure 7B). However, pretreatment of human islets with the PKA inhibitor, H-89, significantly blocked CREB phosphorylation (Figure 7A) and markedly reduced GSK-3 phosphorylation induced by forskolin (Figure 7B). Since Akt can also induce phosphorylation and inactivation of GSK-3, we pretreated human islets with the Akt inhibitor XII to exclude the possibility that PKA induces GSK-3 phosphorylation through activation of PI3K/Akt signaling. Human islets pretreated for 1 h with the Akt inhibitor XII showed significant reduction of Akt phosphorylation at Ser473 (Figure 7C) however, forskolin-induced GSK-3 phosphorylation was not altered in these islets (Figure 7B). These results suggest that activation of cAMP/PKA may engage canonical Wnt signaling through PKA inhibition of GSK-3. However, these results do not exclude that GSK-3 phosphorylation may also be induced through Akt.

To determine whether activation of cAMP/PKA/CREB signaling also engages Irs2/Akt signaling under our treatment conditions, we measured the mRNA levels of the CREB target gene, *Nr4a2*, as well as *Irs2* in response to forskolin treatment. Isolated human islets were treated with forskolin in the presence of 5 or 20 mM glucose for 30 min, 4 and 8 h. Forskolin treatment at either 5 or 20 mM glucose increased *Irs2* mRNA levels 1.3-fold after 30 min and remained 2.4-fold higher than controls after 4 and 8 h (Figure 7D). Treatment with 20 mM glucose alone did not increase the *Irs2* mRNA level. Consistent with the role of forskolin in increasing cAMP levels, the expression of *Nr4a2* was also increased after 30 min (Figure 7D). These data indicate that activation of the cAMP/PKA signaling pathway may activate CREB and promote the expression of *Irs2*, thus engaging the Irs2/Akt signaling pathway.

### Pretreatment of human islets with L-WRN+ to increase proliferation does not diminish subsequent glucose-stimulated insulin secretion or insulin content

**Figure 8:**
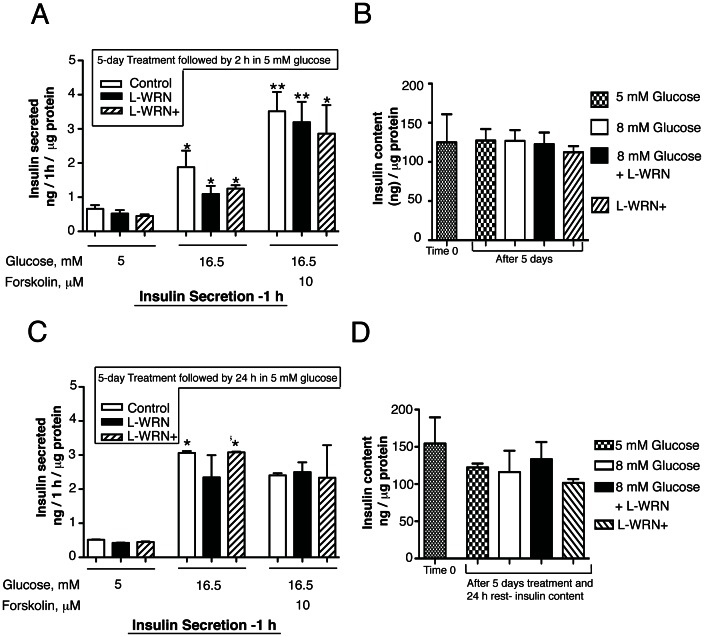
L-WRN+ treatment does not impair insulin secretion. Islets (100) were cultured for 5 days in CMRL-1066 medium containing 10% FBS and 8 mM glucose or in 50% Wnt3a, R-spondin-3 and Noggin conditioned medium (L-WRN) ±8 mM glucose ± Y-27632 and SB-431542 inhibitors as indicated. (A) & (C) Following 5 days incubation, treatments were removed and islets were incubated for 2 h or 24 h, respectively, in cCMRL with 5 mM glucose. Insulin secretion was measured by a static incubation for 1 h in cCMRL containing 5, 16.5 mM glucose ± forskolin. Quadruplicates of 10 islets were assayed. (B) & (D) Insulin content was measured from 20 islets in triplicate at the start of the incubation period (Time 0) and after 5 or 6 days of incubation, respectively, under the conditions shown. Insulin content in islets was extracted by the acid/ethanol method. Insulin secretion and content were detected using Immulite 1000 system and their values were normalized to islet protein. Data are the means ± SE of n = 3 with duplicate samples in each experiment. *P<0.05 and **P<0.01 denote significant differences between the treatment condition and respective control.

In Figure 8A, islets were cultured for 5 days in 8 mM glucose alone or in combination with L-WRN or L-WRN+. The media was removed and islets rested for 2 h in cCMRL containing 5 mM glucose. Islets were then assayed for glucose-stimulated insulin secretion. Insulin secretion was significantly increased in response to 16.5 mM glucose compared to 5 mM glucose, in control, L-WRN or L-WRN+ treated islets. The maximum level of insulin secretion was also determined in response to 16.5 mM glucose in the presence of forskolin to increase cAMP concentrations. In Figure 8B insulin content was assayed at Time 0 and after 5 days treatment with 5 or 8 mM glucose, L-WRN or L-WRN+. There was no significant difference in insulin content between Time 0 (the time the islets arrived from the IIDP center) or following the 5-day treatment period.

In Figure 8C and 8D, this same protocol as described in Figure 8A and 8B was performed, except islets were allowed to recover in cCMRL 5 mM glucose for 24 h. Glucose-stimulated insulin secretion in response to 16.5 mM glucose ± forskolin was increased ∼5–6 fold compared to 5 mM glucose and the insulin content was maintained under all treatment conditions. Collectively, these findings indicate that L-WRN+ treatment significantly increases β-cell proliferation, while preserving human β-cell glucose-stimulated insulin secretion and content.

## Discussion

**Figure 9:**
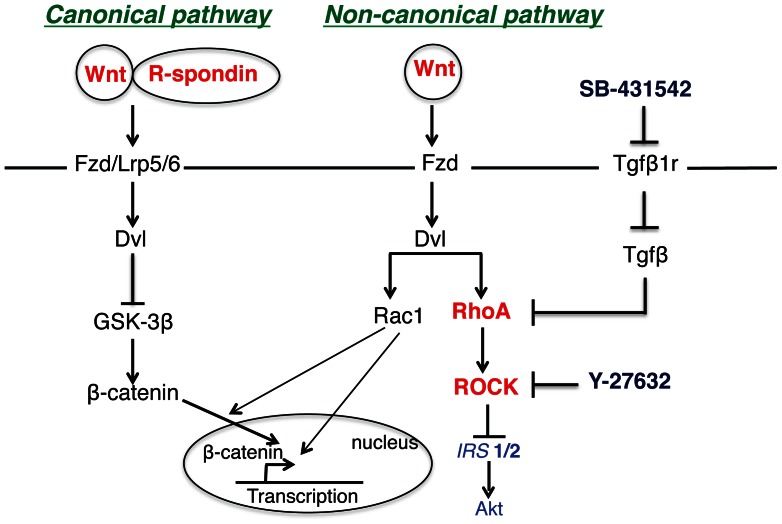
Proposed regulation of Wnt and Rho/ROCK signaling pathways by L-WRN+ in human islets.

In this study, we utilized a novel conditioned medium to unmask the proliferative capacity of β-cells in intact human islets while maintaining the β-cell phenotype. The conditioned medium+ (L-WRN+) expresses elevated levels of Wnt3a, R-spondin-3, and Noggin and contains the ROCK inhibitor (Y-27632) and SB-431542 (that results in RhoA inhibition). As shown schematically (Figure 9), activation of Wnt signaling at the receptor level with L-WRN+ engages both canonical and non-canonical Wnt signaling but also activates RhoA resulting in degradation of IRS1/2 and inhibition of the insulin signaling pathway.

RhoA and its downstream effector, Rho-kinase (ROCK), are involved in the development of insulin resistance, regulation of insulin action and glucose homeostasis [56], [57]. Activation of Rho/ROCK signaling has been shown to increase serine phosphorylation of insulin receptor substrate-1/2 (IRS1/2) leading to its proteasomal degradation and negatively regulates Akt signaling [58], [59]. In contrast, inhibition of Rho/ROCK signaling reduces insulin resistance by increasing the tyrosine phosphorylation of IRS1/2 and Akt phosphorylation [59], [60].

In our approach, we inhibited ROCK signaling directly with Y-27632 [61], [62]. In addition, Rho/ROCK signaling was targeted upstream with SB-431542, a small competitive inhibitor of TGF-βRI superfamily activin-like kinase (ALK) receptors that inhibits TGF-β mediated activation of RhoA, and blocks RhoA-downstream signaling (Figure 9) [63]. Including these inhibitors in our medium (L-WRN+) resulted in the phosphorylation of Akt in human islets, indicating a reduction of insulin signaling pathway resistance (Figure 6B). Chronic hypoxia has been shown to increase signaling through multiple pathways including the RhoA/ROCK pathway [64], [65]. It is probable that the hypoxic conditions associated with the isolation of human islets from cadaveric donors and their shipment upregulates RhoA/ROCK signaling [19], [66].

Further support for the importance of using the combination of L-WRN and RhoA/ROCK inhibitors (L-WRN+), is that treatment of human islets with RhoA/ROCK inhibitors alone only produced a modest increase in DNA synthesis comparable to that obtained with LiCl alone, demonstrating the necessity of the Wnt3a, R-spondin-3 and Noggin components (Figure 2A). In addition, RhoA/ROCK inhibitors did not increase DNA synthesis in combination with LiCl. It is possible that over-inhibition of GSK-3 with LiCl could decrease the phosphorylation of CREB at Ser129 and trigger the dephosphorylation of Ser133. In addition, over-inhibition of GSK-3 may inhibit protein kinases that phosphorylate CREB at Ser133 such as PKA, PKC and p38 [67]. Thus, engaging Wnt signaling at the receptor level in combination with RhoA/ROCK inhibition is required to achieve significant levels of human β-cell proliferation.

An important consideration in the use of L-WRN+ is the maintenance of β-cell phenotype and function. L-WRN+ treatment of human islets results in accumulation of nuclear β-catenin and stimulates the expression of multiple β-catenin target genes (Figure 1C, 5D and 5E) and β-cell specific gene expression (Figure 2C). In addition, L-WRN+ treatment increases gene expression of CREB target genes, *Nr4a2* and *Irs2*, and reduces islet insulin signaling pathway resistance indicated by increased phosphorylation of Akt (Figure 6A and 6B). Although L-WRN+ treatment strikingly increases proliferation, it does not significantly reduce insulin content or alter glucose stimulated insulin secretion. Importantly, the preservation of insulin secretion and content is observed following the removal of L-WRN+, and may well make this strategy applicable to *ex vivo* β-cell expansion prior to transplantation.

This study also provides insights into how L-WRN+ promotes the necessary crosstalk between multiple signaling pathways that regulate proliferation and maintenance of the phenotype of human β-cells, including activation of the insulin/Akt, mTOR, Wnt/β-catenin, PKA/CREB signaling pathways, and inhibition of the RhoA/ROCK pathway. During isolation and shipping, human islets are exposed to stress-related hypoxia. This increases RhoA/ROCK signaling and chronic mTOR activation resulting in insulin resistance. Thus, neither Wnt/β-catenin nor insulin/IGF1 signaling is engaged. Wnt signaling is engaged at the receptor level with Wnt3a and R-spondin-3 that activates canonical and non-canonical Wnt pathways. β-catenin is translocated to the nucleus due to GSK-3 inhibition and in combination with Rac1 signaling [68], [69], increases β-catenin-dependent gene transcription. As we have previously described, appropriate mTOR activity is required for β-catenin nuclear translocation [6]. Non-canonical Wnt pathway activation increases RhoA/ROCK signaling that results in degradation of IRS1/2 and negatively affects Akt phosphorylation. Inhibiting RhoA/ROCK signaling with SB-431542 and Y-27632 reduces the insulin signaling pathway resistance. Our data also suggest that Wnt-mediated DNA synthesis in human islets is PKA dependent, based on H-89 inhibition (Figure 6C and 6D). Furthermore, CREB signaling increases *Nr4a2* and *Irs2* gene expression and increases Akt phosphorylation (Figure 6A and 6B).

In summary, we have used a novel conditioned medium expressing Wnt3a, R-spondin-3 and Noggin to engage Wnt signaling at the receptor level in combination with RhoA/ROCK inhibitors, SB-431542 and Y-27632, to significantly enhance adult human β-cell proliferation and maintain β-cell specific gene expression, insulin secretion and content in intact islets. An advantage of using this conditioned medium is to achieve activation of both canonical and non-canonical Wnt pathways (in a manner that is not possible with exogenous addition of conventional Wnt ligands to culture media) to significantly enhance β-catenin nuclear translocation and proliferative gene expression activity. This study also sheds light on the importance of inhibiting RhoA/ROCK signaling in combination with Wnt signaling to reduce human β-cell insulin signaling pathway resistance. Treatment of adult human islets with this conditioned medium provided highly reproducible effects on proliferation and β-cell specific gene expression independent of the donor's biometrics or origin from different isolation centers. The significance of these findings is that adult human β-cells *in vitro* exhibit substantial levels of proliferation when the proliferative capacity of β-cells is unmasked with appropriate trophic factors, while maintaining the intact islet architecture. Future *in vitro* studies will be conducted to determine the specificity or otherwise of L-WRN+ treatment on the proliferation of β-cells and other islet cell types, in particular α-cells. This study provides new insights into the pathways involved in human β-cell proliferation and the maintenance of the β-cell phenotype and offers a viable method for *ex vivo* β-cell expansion.

## Supporting Information

Table S1 mRNAs regulated by at least 1.5-fold in either direction, assessed by RNA-sequencing, in both of 2 separate islet preparations. Two islet preparations (from different donors) were subjected to RNA-sequencing; within each preparation, subsets of islets were treated with 5 or 8 mM glucose, 8 mM glucose +5 mM LiCl or 8 mM glucose together with L-WRN+. RNA preparation and analysis are documented in the main Methods text. The Table shows those mRNAs regulated similarly in both islet preparations between 8 mM glucose/L-WRN+ and 8 mM glucose alone (fold change of at least 50%, in the same direction); separate columns denote those mRNAs for which changes of this magnitude were observed between 8 mM glucose alone and 5 mM glucose. Read count data (normalized for differences in the total reads obtained for each sample) are given for 5 mM glucose, 8 mM glucose and 8 mM glucose/L-WRN+ conditions for each islet preparation.
(XLS)

Table S2 Gene Ontology and MetaCore process network descriptions for genes upregulated by L-WRN+. Category names are shown together with a p-value denoting the significance of gene over-representation in that category (all p-values shown are significant at a false discovery rate <0.05). Numbers in green text denote the number of genes from the L-WRN+ upregulated dataset; numbers in red denote the total number of human genes that are members of that category. The first tab of the Excel file shows Gene Ontology categorization; the second tab displays MetaCore (Thomson Reuters, http://thomsonreuters.com/products_services/science/systems-biology/) process descriptions.
(XLS)

Table S3 Gene Ontology and MetaCore process network descriptions for genes downregulated by L-WRN+. As for Table S2, but for downregulated genes.
(XLS)

Table S4 Genes of particular functional importance to β-cells and islets in response to L-WRN+ treatment. Two islet preparations (from different donors) were subjected to RNA-sequencing as described for Table S1. The Table shows mRNAs regulated in both islet preparations between 8 mM glucose/L-WRN+ and 8 mM glucose alone. Significantly changed expression (50% change in both preparations, in the same direction) is denoted in bold. Only those genes with directionally similar changes in both islet preparations, or lack of regulation in both islet preparations, are shown. Read count data (normalized for differences in the total reads obtained for each sample) are given for the 8 mM glucose condition. Gene lists are drawn from references as follows: 1) Anderson et al., BMC Dev Biol (2009) 9:65; 2) Kutlu et al., BMC Med Genomics (2009) 2:3; 3) Taneera et al., Cell Metab (2012) 16:122–134.
(XLS)
